# The Role of Eukaryotic and Prokaryotic ABC Transporter Family in Failure of Chemotherapy

**DOI:** 10.3389/fphar.2016.00535

**Published:** 2017-01-10

**Authors:** Raafat El-Awady, Ekram Saleh, Amna Hashim, Nehal Soliman, Alaa Dallah, Azza Elrasheed, Ghada Elakraa

**Affiliations:** ^1^Department of Pharmacy Practice and Pharmacotherapeutics, Sharjah Institute for Medical Research and College of Pharmacy, University of SharjahSharjah, United Arab Emirates; ^2^National Cancer Institute – Cancer Biology Department, Cairo UniversityCairo, Egypt

**Keywords:** ABC family, multiple drug resistance, chemotherapy failure, chemotherapy efflux, p-glycoprotein, BCRP1

## Abstract

Over the years chemotherapy failure has been a vital research topic as researchers have been striving to discover reasons behind it. The extensive studies carried out on chemotherapeutic agents confirm that resistance to chemotherapy is a major reason for treatment failure. “Resistance to chemotherapy,” however, is a comprehensive phrase that refers to a variety of different mechanisms in which ATP-binding cassette (ABC) mediated efflux dominates. The ABC is one of the largest gene superfamily of transporters among both eukaryotes and prokaryotes; it represents a variety of genes that code for proteins, which perform countless functions, including drug efflux – a natural process that protects cells from foreign chemicals. Up to date, chemotherapy failure due to ABC drug efflux is an active research topic that continuously provides further evidence on multiple drug resistance (MDR), aiding scientists in tackling and overcoming this issue. This review focuses on drug resistance by ABC efflux transporters in human, viral, parasitic, fungal and bacterial cells and highlights the importance of the MDR permeability glycoprotein being the mutual ABC transporter among all studied organisms. Current developments and future directions to overcome this problem are also discussed.

## Introduction

Millions of new cases with infectious/malignant diseases are reported every year; many of them may die due to failure of therapy that is mainly attributed to resistance to chemotherapy (Teillant et al., 2015; Crunkhorn, 2016; Saunders and Lon, 2016). Chemotherapy is a broad term that will be utilized in this review to describe chemical agents or drugs used for treatment of different types of diseases caused by many causative microorganisms including virus, bacteria, fungi, parasites, or malignant diseases (Leekha et al., 2011; Wijdeven et al., 2016).

Despite the success achieved in the management of many conditions, we still have a never-ending constant battle against the constantly evolving microorganisms and malignant cells that strive for their own survival (Holohan et al., 2013; Blair et al., 2015; Sanglard, 2016). This is mainly due to resistance, which is a natural phenomenon and defense mechanism created and developed to protect living cells, eukaryotic and prokaryotic, and to maximize their survival.

**FIGURE 1:**
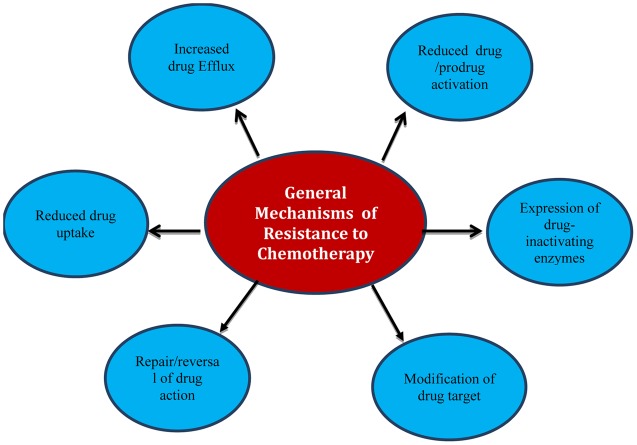
**General mechanisms of resistance to chemotherapy**.

There are a variety of mechanisms that living cells may naturally have (intrinsic) or can develop from their environment or genetic changes (acquired) that give them the adaptation to resist foreign chemicals including chemotherapy (Swanton, 2012; Holohan et al., 2013; Blair et al., 2014; Chen et al., 2014). Drugs are dealt with as foreign toxic material that enters a living cell; therefore they are overcome and counteracted by different ways. Strategies adopted by cells include decreasing the intracellular concentration of chemotherapeutic agents by reducing influx or increasing efflux from cells, inactivation by enzymes and modification of target sites (**Figure 1**).

Despite the existence of resistance mechanisms that are specific to some classes of microbes/drugs, resistance mechanisms that are common among all microbes and many chemotherapeutic agents exist (Borst and Ouellette, 1995; Viveiros et al., 2005; Kanafani and Perfect, 2008; Strasfeld and Sunwen, 2010; Akhdar et al., 2012; Li et al., 2016).

The aim of chemotherapy is to eradicate causative microorganisms or malignant cells. This can be achieved when the chemotherapeutic agent reaches the site of action in the right effective concentration and exerts its pharmacological action.

For a chemotherapeutic agent to reach its site of action, it should traverse many barriers. This is determined by the pharmacokinetics of the drug, which plays the main role in the delivery of the drug to its site of action (Chung et al., 2016; Ramsey and MacGowan, 2016; Yang and Liu, 2016; Zhao et al., 2016). It all starts with the absorption of the drug through lipophilic membranes, followed by distribution to body cells and tissues through the blood. Once the drug reaches the intended target site and concentrates to the therapeutic level, the pharmacological effect is seen in the form of pharmacodynamics – the interaction of the drug with receptors or proteins at the target site causing the body’s response.

A drug may fail achieving biological response due to efflux by the target cells, which is one of the main reasons behind failure of treatment of many diseases.

Efflux of drugs from cells by membrane transporters is the most predominant and mutual mechanism of resistance among all organisms (Tillotson and Tillotson, 2010) These membrane transporters are proteins that belong to a superfamily of genes called the ABC (Holmes et al., 2016; Ye et al., 2016; Zhang et al., 2016). This review sheds light on the different types of ABC gene family, their expression in different microbes/malignant cells, their role as membrane transporters and their role in development of resistance to chemotherapeutic agents. Modulators of the expression or function of the ABC transporter family and new methods to overcome their effects are also discussed.

## The ATP-binding Cassette (ABC)

The ABC is the largest protein transporter superfamily present in all organisms.

This family of genes codes for different proteins (importers and exporters), which translocate a variety of substrates such as sugars, amino acids, ions, peptides, proteins, cholesterol, metabolites and toxins across extra- and intracellular membranes (Linton, 2007; Broehan et al., 2013; Benadiba and Maor, 2016).

They are present in both prokaryotes and eukaryotes and serve many functions. In prokaryotes they represent both influx proteins, which carry nutrients into cells, as well as efflux proteins, which expel toxins and drugs out of the cell. However, in eukaryotes, they are only expressed as efflux transporter proteins protecting the cell from toxins (Higgins, 2001; Breier et al., 2013; Videira et al., 2014).

The common feature shared between all ABC transporters is the fact that they are made of two domains. The first is the NBD and the second is the TMD (Ter Beek et al., 2014). These two domains participate in a coupling mechanism to contribute to the main common function that facilitates import and export (Altenberg, 2004). The NBD catalysis ATP hydrolysis and the resulting energy is used by the TMD to translocate substances through the membrane by conformational changes (Silva et al., 2015). Efflux of drugs by ABC transporters decreases intracellular drug concentration causing failure of chemotherapy. This efflux mechanism is seen in all living cells, prokaryotes and eukaryotes (Holland and Blight, 1999; Sharom, 2008) and will be discussed in depth in each organism.

### Human ABC

In humans, there are 49 known ABC genes classified into seven different families (A–G) depending on their amino acid sequence and eventually their protein domains: ABCA (13 members), ABCB (11 members), ABCC (11 members), ABCD (4 members), ABCE (1 member), ABCF (3 members), and ABCG (5 members) (Dean et al., 2001; Auner et al., 2010). They serve a variety of functions other than drug resistance and they can be expressed as channels, receptors and transporters (Vasiliou et al., 2008).

The members involved in drug efflux from human cells don’t belong to one particular family. There are 12 transporters reported to be responsible of drug efflux, however, three main MDR transporters ABCB1 (P-gp), ABCC1 (MRP), and ABCG2 (BCRP) have a great significance in the efflux of a variety of drugs. Substrates of these three MDR “omnivore” transporters belong to different classes, not just anticancer drugs but include antivirals, antibacterials, antiepileptics, antidepressants, antiparasitics, and antifungals (Sharom, 2008).

The first report about the role of MDR proteins in chemotherapy resistance was released in Ling and Thompson (1974) when Victor Ling and Larry Thompson realized that colchicine failed to enter cytoplasm of a Chinese hamster ovary cell line. Furthermore they found that the same cells were resistant to demecolcine, actinomycin D and vinblastine (Kunjachan et al., 2013).

Expression of efflux transporters in humans is seen in many organs such as the intestine, brain, liver, kidney, adrenals, placenta, and lungs. In the intestine, liver and kidneys they are highly expressed and hinder entry of toxic chemicals including drugs and reduce their bioavailability and alter their pharmacokinetics.

P-glycoprotein and BCRP are present in the epithelia of sensitive tissues like the brain, placenta, and stem cells where they mediate not just MDR, but also multiple drug interaction in the membrane penetration step (Brockmöller and Tzvetkov, 2008; Kunjachan et al., 2013), which may result in toxicity during absorption or secretion. An example is seen in the administration of digoxin with verapamil or quinidine, which compete with digoxin for the P-gp, overcoming it and displacing it from the transporter binding sites, increasing digoxin blood levels. This process can be utilized to increase systemic exposure of orally administered Pgp substrate drugs. This is an example of competition on the absorption level (Lin and Yamazaki, 2003). Similarly, BCRP (ABCG2) is involved in the competitive interactions between MTX (an anticancer drug) and benzimidazoles (antifungal agents), which results in reduced MTX clearance. Again, this kind of interaction can be used to increase drug bioavailability. Interestingly, flavonoids, which are present as flavorings and colorings in food are also substrates exported by ABCG2. Therefore they can be used to overcome ABCG2-mediated resistance. This is an example of competition on the level of secretion (Breedveld et al., 2004; Noguchi et al., 2014).

In addition to their role in drug efflux, ABC transporters are reported to play a role in drug metabolism. Traditional drug metabolism was known to consist of Phase I and Phase II. Phase I is oxidation/reduction of the exogenous compounds to activate pro-drugs or inactivate actives, while Phase II is conjugation of the partially detoxified metabolites. Recently two additional phases were added, called Phase 0 and Phase III of drug disposition. ABC transporters play their role in these phases by modulating the cellular entry and exit of exogenous compounds. In Phase 0, before drugs reach intracellular targets, transporters will control their entry and exit, which results in either augmentation or reduction in pharmacological drug effects. Finally, the role of ABC transporters in Phase III is to assure complete elimination of the metabolized molecules (Brockmöller and Tzvetkov, 2008).

Because ABC members are not just involved in drug resistance, mutations in ABC genes can cause many recessive genetic disorders like cystic fibrosis, neurological disorders, retinal degeneration, cholesterol/bile transport defects, and anemia (Dean et al., 2001). In cystic fibrosis, for example, the cystic fibrosis *trans*-membrane conductance regulator (CFTR/ABCC7) was found to be mutated in many cases (Jordan et al., 2008).

In this review, the main focus will be on the most influential ABC efflux transporters and their role in chemotherapy resistance and failure.

#### Human ABCA Family

Thirteen transporters are reported as members of the human ABCA family. The function and expression status of the most commonly characterized members are presented in **Table 1**. This family of transporters are involved mainly in resistance to anticancer and antiviral agents (Borst et al., 2004; Li et al., 2007; Hedditch et al., 2014).

**Table 1:** Subtypes of ABCA transporters.

Subtype	Expression status	Notes	Reference
ABCA1	Over-expressed in: – Pancreatic ductal adenocarcinomas, – Breast and ovarian cancers, – Cisplatin-resistant human epidermoid carcinoma cells.	– A cholesterol efflux pump in the cell’s lipid metabolism pathway. – Lentiviral knockdown of ABCA1 resensitize chemotherapy-resistant cells.	Fitzgerald et al., 2004; Chou et al., 2011; Mohelnikova-Duchonova et al., 2013
ABCA2	Over-expressed in: – Some human cancer cell lines, – Estramustine- resistant strain of ovarian cancer cell line, – mitoxantrone- resistant small cell lung cancer cell lines.	– Localized in endolysosomal organelle membranes and expressed in many tissues of the central and peripheral nervous systems, macrophages and reproductive tissues (prostate, ovary, uterus). – Antisense targeting of ABCA2 resulted in down-regulation of ABCA2 and re-sensitization to estramustine.	Austen et al., 2003; Davis et al., 2004; Mack et al., 2011
ABCA4	Over-expressed in: – Induced MDR breast cancer cell line.	– The degree of its up-regulation could be used as marker for determining the stage of ovarian cancer and the degree of its resistance to chemotherapy.	Liu et al., 2005; Ween et al., 2015
ABCA5	Over-expressed in: Esophageal – doxorubicin, paclitaxel, and vincristine resistant ovarian cancer cells	– Was first discovered in 2003.	Ohtsuki et al., 2007; Saini et al., 2012; Hlavac et al., 2013

#### Human ABCB Family

##### ABCB1 (P-glycoprotein/MDR1)

**FIGURE 2:**
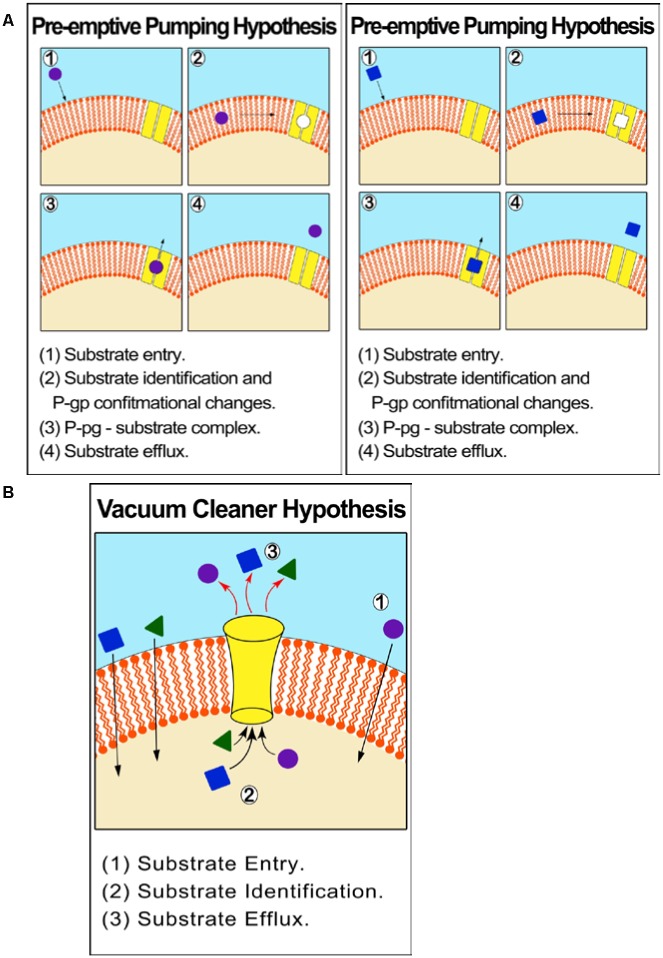
**Proposed mechanism of efflux by ABCB1 (P-gp/MDR1). (A)** Pre-emptive pumping hypothesis: P-gp identifies substrates in the cell membrane and before they reach the cytoplasm. **(B)** Vacuum cleaner hypothesis: P–gp effluxes molecules from within the cytoplasm.

ABCB1 is an atypical membrane transporter found in the kidney, placenta, liver, adrenal glands, intestine and blood–brain barrier and has the most critical and leading role in drug efflux and chemo resistance (Kartner et al., 1983; Higgins, 1992; Kathawala et al., 2015; Silva et al., 2015). P –glycoprotein (P-gp)/MDR1 (ABCB1) was the first discovered efflux transporter and is the most influential in contributing to drug resistance. The relationship between P-gp over expression in tumor cells and drug resistance and failure of chemotherapy is well documented (Veneroni et al., 1994; Gottesman et al., 1996; Duan et al., 2004; Yakirevich et al., 2006; Ween et al., 2015). The P-gp mechanism of efflux is unclear but can be described by two different hypotheses: One suggests that P–gp effluxes molecules from within the cytoplasm (vacuum-cleaner hypothesis) while the other from within the cell membrane. The latter hypothesis is supported by theoretical models and empirical *in vitro* experiments, which show that P-gp identifies substrates before they reach the cytoplasm (‘preemptive pumping’). Once this is accomplished, the P-gp activates conformational changes resulting in ‘induced-fit’ interaction with a wide range of MDR substrates (**Figure 2**).

Nevertheless, both hypotheses can be supported by the fact that P-gp is over-expressed in the barrier forming plasma membrane, explaining its protective mechanism toward the cell. An experiment on Chinese hamster ovary cells proved that P-gp constituted about 20% of the total plasma membrane proteins intrinsically (Ferreira et al., 2015).

A six to sevenfold increase in P-gp expression in the tumors of five patients with lung metastasis after chemoperfusion of doxorubicin for only 20 min was reported (Abolhoda et al., 1999). This phenomenon of up regulation of efflux transporters as a result of chemotherapy is known as acquired resistance. This type of resistance also refers to cases where MDR proteins appear in tumors of tissues where they are normally not present like in gliomas (Kirtane et al., 2013; Kunjachan et al., 2013). Drug efflux is not the only way of conferring resistance in P-gp over expressing cells. P-gp over expressing cells are less sensitive to different kinds of caspase-dependent cell death, including those mediated by Fas ligand (apoptosis- inducing TNF) and serum withdrawal. This means cells with high P-gp expression may have a higher survival rate or less apoptosis induction (Ruefli et al., 2000).

Another dimension of development of MDR by cancer cells is the transfer of functional P-gp from P-gp positive to P-gp negative cells both *in vitro* and *in vivo* (Levchenko et al., 2005). The transfer can happen by means of extra cellular vesicles such as exosomes explaining how some sensitive cells acquire drug resistance (Kirtane et al., 2013).

Over expression of ABCB1 in cancer cells results in resistance to drugs from different classes such as vinca alkaloids, taxanes, anthracyclines, epipodophyllotoxines. A study using Paclitaxel on Abcb1 knockout mice cells, showed accumulation of the drug in their gastrointestinal tract and brain. This shows that ABCB1 is responsible of paclitaxel’s elimination into the bile and for preventing it from crossing the blood–brain barrier (Schinkel et al., 1996; Kirtane et al., 2013).

##### Ionizing radiation and P-glycoprotein

Radiotherapy represents one of the main pillars of cancer therapy. It plays a major role in definitive, adjuvant and palliative treatment. It kills cells by damaging the DNA, changing genes and inducing cell apoptosis. Being non-selective and lethal, the dose of radiation must be limited to minimize damage to healthy cells. Just like in chemotherapy exposure, upregulation of MDR efflux transporters like MDR1 (P-gp) happens in response to radiotherapy (Kim et al., 2012; Wang et al., 2012). A study using squamous cell carcinoma cell lines, T-167 and T-409, exposed to different doses of ionizing radiation showed overexpression of P-gp after exposure of cells to 2 Gy. This information means exposing cancer cells to ionizing radiation before pharmacotherapy may result in chemotherapy resistance. This fact can be utilized in combinatorial strategies of cancer therapy to optimize doses and regimens and to promote using ionizing radiation preferably after chemotherapy to reduce the risk of resistance (Shareef et al., 2008).

##### Polymorphism of ABCB1

Several SNPs in the ABCB1 gene were reported and were shown to modulate resistance/response to therapy (Gréen et al., 2006; Obata et al., 2006; Kim et al., 2009; Bergmann et al., 2011; Peethambaram et al., 2011; Ween et al., 2015). This is because protein expression or function is altered, thereby resulting in differences in pharmacology and response to ABCB1 inhibitors. For example, polymorphisms in the ABCB1 and ABCG2 genes in AML patients may contribute to different survival outcomes and toxicities due to decreased drug efflux in AML blasts and normal progenitors. If the patient’s cancer cells have a limited ABCB1 expression and function, using an inhibitor could increase toxicity to the normal bone marrow compartment while having little impact on the leukemic cells, this means more harm than benefit (Shaffer et al., 2012). Therefore, the goal of studying SNP is a better understanding of inter-individual pharmacologic variation due to polymorphism of ABCB1, which may open a window for individualized therapy based on the patients ABC transporter genotype (Hamidovic et al., 2010; Diaz-Padilla et al., 2012).

##### ABCB1 inhibitors

Competitive inhibitors of ABCB1 are categorized into three generations of drugs based on pharmacodynamics characteristics (Kathawala et al., 2015): First-generation ABCB1 inhibitors, including verapamil and CCBs, non-CCBs such as flupenthixol, chlorpromazine, CSA, quinine and quinidine (Abdellah et al., 2015). Second- generation inhibitors such as valspodar and third generation such as tariquidar and zosuquidar are under clinical investigation (**Table 2**). Unfortunately, many of the ABCB1 inhibitors may also inhibit cytochrome P450 and impair drug clearance, putting a risk of toxicity and adverse effects to normal cells (Pusztai et al., 2005; Kathawala et al., 2015; Li et al., 2015).

**Table 2:** Inhibitors of ABCB1 (P-gp/MDR1).

Class	Examples	Reference
First generation	(a) CCBs (Verapamil), (b) Non-CCBs (flupenthixol, chlorpromazine, CSA, quinine, and quinidine)	Tsuruo et al., 1981; Pennock et al., 1991; Bechtel et al., 2008; Kelly et al., 2011; Koski et al., 2012; Abdellah et al., 2015
Second generation	Dexverapamil Valspodar Biricodar citrate	Wilson et al., 1995; Tidefelt et al., 2000; Minderman et al., 2004; Amiri-Kordestani et al., 2012
Third generation	Tariquidar, Laniquidar and zosuquidar Elacridar Mitotane and Annamycin	Consoli et al., 1996; Kemper et al., 2004; Fox and Bates, 2007; Dorner et al., 2009; Li et al., 2015
Natural products	Flavonoids from Chinese plants Neochamaejasmin B Cytarabine and halaven	Michalak and Wesolowska, 2012; Huang et al., 2014; Lopez and Martinez-Luis, 2014; Pan et al., 2015; Long et al., 2016
Monoclonal antibodies	MRK-16 MRK-17	Pearson et al., 1991; Naito et al., 1993
Others	Gatifloxacin Celecoxib	Kwatra et al., 2010; Chae et al., 2015

Alteration of pharmacokinetics by ABCB1 inhibitors may necessitate decreasing chemotherapeutic doses to prevent toxicity. However, this would be sacrificing the benefit from the inhibition. Designing potent MDR inhibitors free from unwanted pharmacological effects is the ultimate goal. Drugs like CSA and Verapamil are good inhibitors of ABCB1/ABCB4 and therefore it would be a strategy to saturate MDR1/MDR3 transporters to increase intracellular concentrations of drugs (Amiri-Kordestani et al., 2012).

A phase III trial by Southwest Oncology Group (SWOG) supported the combination of CSA with induction chemotherapy, as it results in improvement in 2-year overall survival. Fortunately, further studies on leukemia patients proved that administering CSA with treatment improved overall survival from 4 to 12 months. However, there was no correlation between chemotherapy dose and level of P-glycoprotein expression (Shaffer et al., 2012).

An investigation to test the interaction of fourth generation fluoroquinolones–gatifloxacin with the three dominant efflux pumps: P-gp, MRP2, and BCRP was done and its results showed that cellular uptake of erythromycin in cells with P-gp overexpression was increased in the presence of quinidine or gatifloxacin.

This shows that quinidine and gatifloxacin are also substrates and can be used as inhibitors of P-gp. Further testing showed that quinidine prevents erythromycin efflux by non-competitive inhibition of P-gp whereas gatifloxacin appears to cause competitive inhibition. The indirect inhibition of P-gp and MRP2- mediated efflux by gatifloxacin is through ATP inhibition and fluoroquinolones were proven not to be substrates of BCRP. Knowing that P-gp in bacteria is ATP-dependent means that inhibitors can be developed to prevent such ATP dependent efflux, fighting antibacterial resistance (Kwatra et al., 2010).

Moving to newer agents, Tariquidar’s effect as an inhibitor was investigated in advanced breast cancer and was found to reverse resistance to doxorubicin, vinblastine, and paclitaxel (Walker et al., 2004; Amiri-Kordestani et al., 2012). Another clinical trial on non-small cell lung cancer (NSCLC) patients, however, was suspended because it resulted in toxic effects and poor responses. Still, those contradicting results don’t change the fact that Tariquidar is a potent effective inhibitor and a lead compound with many analogs (Li et al., 2015).

Another interesting study of MDR inhibition in cells over-expressing COX-2 and MDR-1 has shown that celecoxib, a COX-2 specific inhibitor, induces apoptosis and down-regulation of MDR-1 expression (Arunasree et al., 2008; Chae et al., 2015). This indicates that celecoxib can be considered a potential MDR inhibitor.

##### ABCB4

ABCB4 (MDR3) is very similar to the ABCB1/MDR1/P-gp in structure (Arai et al., 1997). ABCB4 protein is up-regulated in soft tissue sarcomas (Januchowski et al., 2013; Mohelnikova-Duchonova et al., 2013) and increased levels are associated with a weak response to chemotherapy (Duan et al., 2004). It was found to be up-regulated 16-times in paclitaxel resistant ovarian cancer cell lines compared to parental cell lines. siRNA knockdown of ABCB4 resensitizes cancer cells to paclitaxel. ABCB4 was up-regulated 176-fold in doxorubicin- and 147-fold in vincristine-resistant sublines of ovarian cancer cells which proves that ABCB4 does contribute to chemotherapy resistance just like ABCB1 (Duan et al., 2004; Morita and Terada, 2014).

##### ABCB11

Till 2007, few reports were published about ABCB11. However, latest researches proved that ABCB11 is the bile salt exporter protein (BSEP). Mutations in ABCB11 which causes deficiency of ABCB11 increase the risk of liver cancer (Strautnieks et al., 2008). Its role in chemoresistance is not well studied (Childs et al., 1998). ABCB11 research has so far been focused on liver disease (Ween et al., 2015).

#### Human ABCC Family

The discovery of ABCC as an efflux pump goes back when some researchers reported that some lung cancer cells not expressing P-gp show resistance to MTX, doxorubicin, etoposide and vincristine due to increased efflux of drugs (Cole et al., 1994; Grant et al., 1994). This led to the discovery of ABCC1 (MRP1). These transporters have broad spectrum anticancer resistance activity, making them part of the three main MDR proteins. MRP1 specifically, has a great abundance in primary ovarian tumor cells. A high *MRP*1 expression in primary neuroblastoma was strongly correlated with poor patient outcome. However, development of inhibitors of these MDR transporters isn’t advancing much and the number of compounds that enters clinical trials is limited, one of them is sulindac (Burkhart et al., 2009). Other members of the ABCC family include ABCC2, ABCC3, ABCC4, ABCC5, ABCC6 and 12 (Ween et al., 2015). Overexpression of these efflux pumps was shown to be associated with resistance to many anticancer drugs such as cisplatin, etoposide, doxorubicin, vincristine, MTX and purine analogs (Baiceanu et al., 2016). Protein expression of ABCC10 and ABCC11transporters was shown recently to be associated with survival of colorectal cancer patients (Krizkova et al., 2016).

#### Human ABCG Family

##### ABCG2 (Breast cancer resistance protein/BCRP/MXR)

The first report about a possible link between ABCG2 expression and prognosis of leukemia was mentioned in 1997 (van den Heuvel-Eibrink et al., 2007). This was followed by a debate due to contradicting results regarding a correlation between ABCG2 and response to chemotherapy (Maliepaard et al., 1999; Doyle and Ross, 2003; Sparreboom et al., 2005). The possible reasons behind the contradicting results are that: first, the drugs used in some studies were poor substrates to the protein itself. Second, mRNA levels that were used to detect expression may not reflect correct levels of protein expression or activity. Third, design of some studies might be poor or inaccurate (Mao and Unadkat, 2014). Studies show that the same inhibitors used against P-gp like the latest Tariquidar derivatives can be used for both BCRP and P-gp (Li et al., 2015).

##### Exosomes and ABC

Exosomes are nano sized (50–100 nm) lipid bilayer/membrane-bound vesicles generated and released from the cell interior. Exosome formation is a natural process, and they are considered as the “cargo” or “garbage bags” either excreting wastes or transporting molecules between cells. Their role in cancer is seen as intercellular communication, promotion of signal transduction as well as the transfer of membrane receptors, proteins, mRNA, and miRNAs between cells and this contributes to tumorigenesis, metastasis, and chemo-resistance (Urbanelli et al., 2013).

In addition, exosomes transfer messages from tumor cells to immune and stromal cells. This contributes to the development of the tumor niche and freedom from immune system. One of the problematic proteins that can be transferred by exosomes from cell to cell is P-gp leading to acquired resistance to chemotherapy among cancerous cells. The second way by which exosomes mediate drug resistance in cancer is by encapsulation of chemotherapeutic agents intracellularly and their excretion out of the cell (Zhao et al., 2015).

Claire Corcoran and colleagues developed docetaxel-resistant prostate cell lines in 2012 and results reflected that one of the ways cells acquired resistance was due to the transfer of P-gp by exosomes. This acquired resistance to Docetaxel also resulted in cross-resistance to a range of other anticancer drugs including anthracyclines. Furthermore changes in metastasis characteristics like invasion, motility, migration, proliferation, and growth were developed (Corcoran et al., 2012).

### Bacterial ABC

Numerous mechanisms of antibacterial resistance exist and threaten the activity and efficacy of antibiotics. These include enzymatic inactivation of drugs, drug target alteration and reduced transport of antibiotics to the bacterial cells. However, increased active efflux of drugs is considered the major problem because expression of one species of multidrug efflux pumps can result in resistance to a variety of antibiotics (Robertson et al., 2005).

Bacterial cells, like mammalian cells, also express different families of ABC transporters that also play a role in resistance to antibiotics (Davidson et al., 2008). After the revolutionary discovery of efflux systems in humans in 1972 by Dano, prokaryotes ABC studies began through the analysis of histidine/maltose uptake systems in *S. enterica* and *E. coli*. Starting from 1978, Levy and McCurry hit the road to research about antibiotic resistance by efflux from bacterial transporters by discovering the first bacterial efflux transporter.

Three functional classes of ABC systems exist in bacteria: Importers, exporters and the third is involved in translation of mRNA and in DNA repair (Higgins et al., 1986; Locher, 2016).

Some bacterial transporters are specific for certain substrates while others provide MDR as a broad-spectrum resistance. Bacterial MDR transporters play a role in the survival of bacteria not just by effluxing toxic chemicals but they are also involved in infections by exporting virulence factors, which eventually result in colonization and infecting mammalian cells (Lubelski et al., 2007; Murphy et al., 2016).

Bacterial transporters contributing to MDR can be classified into five groups: the ABC superfamily, MFS, the MATE family, the SMR, and the RND superfamily (Du et al., 2015).

Adenosine triphosphate-binding cassette-type MDR transporters play a role in gram-positive bacterial drug resistance, such as in *E. faecalis* and *S. pneumoniae*. However, ABC doesn’t have the greatest role in prokaryotic drug resistance compared to other MDR systems (Hürlimann et al., 2016). Actually, the MFS efflux transporters are most dominant in gram positive bacteria whereas in gram negative bacteria, the RND system is the most prevailing (Sun et al., 2014).

The first ABC type MDR bacterial transporter discovered is the *lmrA*, which is a protein that is homologous to the human P-gp (Lubelski et al., 2007). The role of *lmrA* in antibiotics resistance was confirmed in a study using a drug-hypersensitive strain of *E. coli* where overexpression of *lmrA* in this bacteria lead to acquired resistance to antibiotics like aminoglycosides, lincosamides, macrolides, quinolones, streptogramins, tetracyclines, and chloramphenicol (Lubelski et al., 2007).

*Streptococcus pneumoniae* has over 60 ABC transporters (Sun et al., 2014). Most of them are importers required for the uptake of various nutrients. Only a few ABC transporters export toxic agents and antibiotics. Many ABC transporters have important effects on pathogen–host interactions, either by improving bacterial growth in nutrient-restricted conditions, wider effects on bacterial physiology, or by reducing sensitivity to host antimicrobial peptides or to antibiotics (Durmort and Brown, 2015).

### Fungal ABC

Fungi are eukaryotic organisms, they share a lot of similarities with mammalian cells, which means antifungals have a low drug selectivity compared to other drug classes, restricting the therapeutic options for treating fungal infections.

Antifungal therapy gets more complicated as drug efflux through membrane transporters contributes to MDR. The main two MDR efflux transporter systems in fungi are the ABC and MFS (Cannon et al., 2009). ABC systems in fungi have a greater role in drug resistance due to the broad spectrum of substrates compared to the more specific MFS (Holmes et al., 2016). This generates a greater problem especially in immune-compromised patients experiencing opportunistic infections (patients undergoing chemotherapy, transplantation, HIV patients; Ahmad et al., 2013).

Efflux of broad spectrum antifungal drugs such as Azoles plays a critical role in resistance of *Aspergillus nidulans*, *C. albicans*, and *S. cerevisiae* (Del Sorbo et al., 2000). *Candida* species and *Aspergillus* are the two most deadly fungal infectious pathogens. Fifty to sixty percentage of the opportunistic infections are caused by *C. albicans* –Candidiasis.

#### Candida

Superficial infections caused by *C. albicans* are best treated with azoles whereas the more severe fatal systemic infections are usually treated with triazoles and recently echinocandins. Resistance by drug efflux is one of the four major mechanisms of resistance in candida. *C. albicans* show an overexpression of three efflux pumps: CDR1 (Cacdr1p) and CDR2 (Cacdr2p), both are ABC transporters in addition to CaMdr1p (MFS transporter) resulting in Azole resistance (Cannon et al., 2009; Bhattacharya et al., 2016; Tsao et al., 2016).

A recent study was undertaken to identify the role of efflux pumps in azole resistance in clinical isolates of *C. albicans*. Thirty isolates susceptible to amphotericin B and 5-fluorocytosine were collected from different non-HIV-infected patients in four hospitals in Shanghai. Rhodamine 6G was used to detect the efflux activity by flow cytometry analysis. Twelve *C. albicans* isolates showed resistance to at least one kind of triazoles. It was found that azole-resistant isolates had more efflux pump activity, as well as overexpression of CDR1 and CDR2 (ABC genes), proving that these ABC efflux systems are the reason behind Azole resistance in *C. albicans* (Liu et al., 2015).

In another experiment to understand resistance of candida to Azole drugs, recombinant forms of *C. albicans* efflux transporters were expressed in *Saccharomyces cerevisiae*. The strains expressing the transporters had a higher resistance to Azoles compared to the parent strain. In the same study rhodamine 6G was used with HTS (high throughput screening) to check the efflux of 1200 drugs through the pumps. Nine compounds were found to be substrates of *C. albicans* efflux pumps and the monoamine oxidase inhibitor clorgyline was found to successfully inhibit both ABC transporters CaCdr1p and CaCdr2p. It reversed the resistance to fluconazole not just in *C. albicans* but in *C. glabrata* too. It also acts on the MFS transporter. This means it is an important lead drug that can be used to optimize reversing drug resistance in fungal ABC systems, especially azole resistance in *Candida* (Holmes et al., 2012).

Results of an investigation using two antibodies and specific pump inhibitors in 18 *C. albicans* isolates showed that there is a higher overexpression of Cacdr1p (2- to 20-fold) compared to Cacdr2p in resistant strains. The result emphasizes the greater influence and contribution of Cacdr1p compared to CaCdr2p in resistance and antifungal therapy failure (Holmes et al., 2008; Piecuch and Obłak, 2014).

#### Aspergillus

*Aspergillus* contributes to opportunistic infections in immunocompromised patients and resistance is a continuous problem. Triazole resistance in *Aspergillus* fumigatus was first described in 1997 and has increased in frequency over the last decade. The same efflux pumps and mechanisms of *Candida* apply to *Aspergillus* (van der Linden et al., 2011).

### Viral ABC

Antiviral drug resistance is composed of two subdivisions: human’s cellular resistance to antivirals, and mutations in Tyrosine Kinase of the virus itself. Viruses lack any transporters because they depend on the host’s cellular mechanisms and machinery for replication and survival. Human cellular resistance to antivirals follows the same principles of MDR. P gp, the playmaker of MDR is a major cause of failure of therapy in viral infections (Hernandez et al., 2013).

Most antiviral drugs suffer resistance mainly due to the fact that they are substrates of P-gp (Silva et al., 2015). Resistance is mostly seen in immunocompromised patients taking antivirals for a prolonged period of time. In such patients, expression of efflux transporters is increased. A study was conducted using the protease inhibitors ritonavir, indinavir, saquinavir, and nelfinavir in human lymphocyte cell lines overexpressing MDR1 and MRP1 genes. The effect on the uptake of those drugs was found to be negative. Lower concentrations of the drugs were found inside the lymphocytes with higher transporter densities. The second part of the test used the same drugs along with potential MDR inhibitors for both P-gp and MRP1 (GF 120918 and MK 571) and revealed intracellular concentrations that are equal to normal non-resistant cell lines (Jones et al., 2001).

The role of drug efflux transporters (P-gp) in failure of HIV treatment with atazanavir was demonstrated by Robillard et al. (2013) who showed that mdr1a^-/-^, mdr1b^-/-^, and Abcg2^-/-^ triple knockout mice accumulate more atazanavir in their brain and testes compared to their wild-type counterparts. They also showed that the P-gp inhibitor elacridar increases the concentration of atazanavir in the brain and testes of wild-type mice compared to vehicle-treated mice.

Viral hepatitis is another type of viral infection that suffers resistance problems with pharmacotherapy. For hepatitis B and C, Sofosbuvir, an oral drug approved by the FDA in 2013, have been shown to be a substrate of P-gp and BCRP. Co-administration of sofosbuvir with intestinal P-gp inducers like rifampin and St John’s Wort decreases Sofosbuvir’s plasma concentration and therefore decreases it’s therapeutic effect. It is, therefore, recommended to use this drug with P-gp or BCRP inhibitors to enhance its intracellular concentration (Devi and Locarnini, 2013).

### Parasite ABC

Controlling the spread of many parasitic infections is more difficult than bacterial/viral infections because of the lack of vaccines available, the slow development of newer agents and the fast emergence of resistance. Resistance in parasites involves two important transporter-dependent mechanisms. The first mechanism is through decreased drug uptake into the cell due to the loss/down-regulation of a transporter required for uptake. This type is associated with resistance to arsenicals and diamidines in African trypanosomes. The second and more relevant type of resistance is the prevalent ABC transporters efflux (P–glycoprotein) (Borst and Ouellette, 1995).

Parasites are eukaryotes and they are therefore expected to share a lot in common with mammalian cells. This includes sharing the same set of ABC genes. Luckily, being eukaryotic and similar to human transporters this helps in the research field regarding resistance. Even the nomenclature is not much different. The homolog of mammalian P-gp, for instance, in *Entamoeba histolytica* is known as EhPgp1. Furthermore it was found that within the different classes of parasitic species there are some differences in the ABC transporters, conferring different resistance toward different drugs. For example comparative studies between *Leishmania* and *Trypanosoma* ABC gene families showed differences in the genes, resulting in differences in resistance too (Yernaux et al., 2006). Examples of resistant parasites include the emetine- resistant *Entamoeba* spp., mefloquine-resistant *Plasmodium* spp., and antimonials- resistant *Leishmania* spp. Latest studies on resistance in parasites mainly focused on *Plasmodium falciparum*, *Leishmania*, and Nematodes (Ponte-Sucre et al., 2013).

#### Malaria

*Plasmodium falciparum* is one of the five plasmodium protozoa species and is associated with the highest morbidity and mortality of the infectious disease –Malaria. Resistance to antimalarials creates a great hurdle toward treatment. Mefloquine was a successful 1st line drug against this parasite in 1984 but unfortunately resistance was developed within 6 years. Back then; it wasn’t clear how this resistance developed. Later, it was proved that resistance to this drug was due to up-regulation of the PfMDR1 gene also known as Pgh1 (Reamtong et al., 2015; Kaewpruk et al., 2016).

Overuse and wrong prescribing patterns of chloroquine, a drug from the same class, shows increased patterns of resistance due to mutations in Pfmdr1/P-gp as well as *P. falciparum* chloroquine resistant transporter (PfCRT). PfCRT has a dual role in Chloroquine resistance. It is a transporter that effluxes Chloroquine and transports Glutathione (GSH) by influx to displace Chloroquine from the Heme group, leading to failure of therapeutic effect. According to latest genetic studies that used the frequency of mutations as resistance markers, the higher the number of mutations in the Pfmdr1 and PfCRT genes, the more likely the species will develop resistance (even in combinatorial therapy), making treatment more complicated (Patzewitz et al., 2013).

PfATP4 is a newly discovered cation P-type transporter that is responsible of maintaining low cystolic Na+ concentrations. New antimalarial drugs belonging to different chemical classes like spiroindolones, pyrazoles, and dihydroisoquinolones were found to disrupt Na+ concentrations inside the cell. Resistance toward these new agents unexpectedly happened due to mutations in the PfATP4 transporter. The fact that this transporter develops mutations in turn resulting in resistance is an important interesting topic of research because its original function (homeostasis of cystolic Na+) is devoid of any efflux or chemoresistance. With the latest findings of its role in antimalarial resistance, this unique transporter could open a window to direct research toward developing new antimalarial drugs (Spillman and Kirk, 2015).

#### *Leishmania*sis

*Leishmania*sis is a parasitic disease caused by the bite of *Leishmania* infected Phlebotomine sandflies. This parasitic disease is very serious and prevalent in developing countries yet it is considered one of the worlds most ignored diseases (McCall et al., 2013). It is the second serious parasitic infection after malaria worldwide (WHO, 2010).

Multidrug resistance (MDR) is present in *leishmania*sis and as expected, ABC transporters are a major factor (Grebowski et al., 2016). Overexpression of the membrane-bound ABC transporters MDR associated protein 1 (MRP1) and P-gp (on the surfaces of *leishmania*s) is one of the mechanisms of antimonial resistance (Mandal et al., 2009).

The first-line treatment for more than 70 years for *leishmania*sis was pentavalent antimonials (Sb). However, *leishmania*sis treatment is poorly developed due to the limited number of agents available. Furthermore resistance to antimonials results in treatment failure and increasing doses of the drugs doesn’t stop the occurrence of resistance (Haldar et al., 2011). Techniques and agents to counteract resistance are under development. For example, Lovastatin is an agent that was found to inhibit both MRP1 and P-gp, allowing accumulation of antimonials (Sb) intracellularly (Mookerjee Basu et al., 2007).

Also, another study on sodium antimonial gluconate (SAG) resistant-*leishmania* was done by coadministering Glycyrrhizic acid (GA) with SAG. Results showed that this combination achieved higher concentrations of SAG inside the parasitic cell. This is due to the suppression of MRP1 and Pgp by GA. GA (liquorice extract) has another advantage of being a cheap readily available agent in comparison to other agents (Bhattacharjee et al., 2015).

#### Nematodes

Nematodes have more ABC systems genes and greater diversity than mammals including class 1 and class 2 ABC systems/transporters. They have the same structure like the mammalian ABC systems, made of two domains and play the same MDR role in chemotherapy. Recent genetic studies showed an association between anthelmintic selection and ABC transporters by comparing between resistant and non-resistant populations of parasitic nematodes (Ardelli, 2013).

Overexpression of ABC transporters, specifically P –gp occurs in drug resistant cells post-drug exposure. Deletion/ modification of ABC genes in mice, mainly P-gp and the multidrug resistance associated protein (MRP), enhanced sensitivity to drugs, particularly ivermectin. A recent study focused on comparing nematodes resistance to ivermectin and moxidectin by experimenting the effect of P-gp inhibitors against the two macrocyclic lactones. Investigations proved only a partial degree of cross resistance (Ardelli, 2013). Fortunately, MDR reversing agents/inhibitors like verapamil used in mammalian cells successfully prevent resistance in parasites too.

## Non-Traditional Strategies to Overcome Effects of ABC Transporters

**FIGURE 3:**
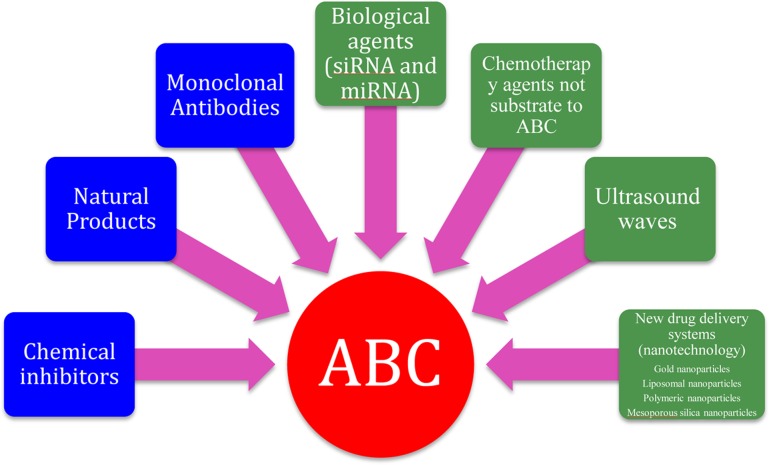
**Strategies for inhibition of ABC transporters.** The blue boxes represents traditional methods to overcome ABC transporters while the green boxes represents non-traditional methods.

In addition to the use of chemical agents, monoclonal antibodies and natural products (mentioned earlier in this review) to reverse the resistance mediated by ABC transporters, there are now non-traditional methods evolving to overcome problems associated with traditional ABC transporter inhibitors. These strategies include the use of biological agents such as RNA interference and microRNA mimetics to down-regulate the expression of ABC family members (Shen et al., 2013; Kasinathan et al., 2014; Guo et al., 2015), development of new drug delivery systems that can bypass the ABC family of transporters such as nanoparticles (Callaghan et al., 2014; Lee et al., 2014; Singh et al., 2015; Bar-Zeev et al., 2016; Yang et al., 2016), development of new chemotherapeutic agents that are not substrate to ABC transporters (Su et al., 2012) and the use of ultrasound waves (Wu et al., 2011) (**Figure 3**).

## Conclusion and Future Perspectives:

In summary, the ABC gene superfamily represents many proteins that perform a variety of vital cell functions including influx and efflux in prokaryotes, while only efflux in eukaryotes. Influx through ABC transporters seen in prokaryotes, such as bacteria, transports important nutrients and molecules into the cell. Efflux of toxic foreign chemicals by ABC proteins happens in all organisms and it’s a natural protective phenomenon. However, it results in failure of chemotherapy by reducing intracellular drug concentrations. In mammals, three dominant ABC efflux proteins result in chemotherapy failure: P-gp (MDR1), breast cancer resistant protein (BCRP) and the MRP1. Overexpression of these transporters results in either specific or MDR and also affects the success rate of different combinatorial therapies.

In fungi and parasites, proteins similar to the human ABC transporters were found. In bacteria, however, ABC system doesn’t seem to be the most influential. It is one of five different systems responsible of chemotherapy resistance. Furthermore, there are differences between gram-positive and gram-negative bacterial drug resistance, because different gene families dominate in each. Research regarding chemotherapy resistance mediated by such efflux transporters is a never-ending process. Investigating the different transporters, their structure, function and most importantly their role in drug resistance helps in optimizing drug development to overcome chemotherapy failure. Instead of wasting money, time and effort in developing newer agents that suffer the same resistance problem, focus should be directed toward tackling the already existing drug resistance. Methods used to tackle resistance by ABC transporters are divided into four main strategies: drugs that specifically target resistant cells, novel nanotechnologies to provide high-dose, targeted delivery of chemotherapy, compounds that interfere with non-genomic transfer of resistance, and approaches to reduce the expression of P-gp within tumors such as ultrasound exposure that has been found to down-regulate efflux transporters.

Despite considerable *in vitro* success of MDR protein inhibitors, there are no compounds currently available to “block” P-gp–mediated resistance in the clinic. The failure may be attributed to toxicity, adverse drug interaction, and numerous pharmacokinetic issues.

More attention should be directed toward designing potent MDR inhibitors free from unwanted pharmacological effects to achieve higher intracellular drug concentrations and better therapeutic outcome. In the future, ABC transporters may be utilized and targeted to treat diseases and to increase selectivity toward disease-causing organisms.

## Author Contributions

RE-A: Idea, design, writing and submission of the article. ES: Formulation and revision of the manuscript. AH: Collection and review of literature. NS: Collection and review of literature. AD: Collection and review of literature. AE: Collection and review of literature. GE: Collection and review of literature. All authors approved the final version of the manuscript.

## Conflict of Interest Statement

The authors declare that the research was conducted in the absence of any commercial or financial relationships that could be construed as a potential conflict of interest.

## Abbreviations

ABC: ATP binding cassette
AML: acute myelogenous leukemia
BCRP: breast cancer resistance protein
*C. albicans*: *C. albicans*
CCB: calcium channel blocker
CFTR: cystic fibrosis *trans*-membrane conductance regulator
COX-2: cyclooxygenase-2
CSA: cyclosporine A
*E. coli*: *Escherichia coli*
*E. faecalis*: *Enterococcus faecalis*
MATE: multidrug and toxic compound extrusion
MDR: multiple drug resistance
MFS: major facilitator superfamily
miRNA: micro RNA
MRP: multiple resistance protein
MTX: methotrexate
NBD: nucleotide binding domain
pfMDR: plasmodium falciparum multiple drug resistance
P-gp: P-glycoprotein
RND: resistance-nodulation-division
siRNA: small interference RNA
SMR: small multidrug resistance
SNP: single nucleotide polymorphism
TMD: *Trans*-membrane domain
